# Deep Sclerectomy: Safety and Efficacy

**DOI:** 10.4103/0974-9233.56223

**Published:** 2009

**Authors:** Zsolt Varga, Tarek Shaarawy

**Affiliations:** From the Service d'Ophtalmologie, Hôpitaux Universitaires de Genève, 22 rue Alcide Jentzer, 1211 Genève, Switzerland

**Keywords:** Deep Sclerectomy, Nonpenetrating Glaucoma Surgery

## Abstract

Deep Sclerectomy is a non penetrating surgical procedure for the treatment of open angle glaucoma. In this article we will describe the surgical technique, the indications for surgery and will review the scientific literature on surgical outcome following this procedure. We will also discuss the important role played by antimetabolites, implants and the use of gonipuncture to achieve the desired IOP reduction.

## INTRODUCTION

Over the past decades, non penetrating surgical procedures have been developed to improve the safety of glaucoma surgery. Deep sclerectomy (DS) has emerged as one of the more established non penetrating procedures and a growing body of evidence on its safety and efficacy has become available. Following DS, the aqueous outflow is enhanced by removing the inner wall of Schlemm's canal and juxta-canalicular trabecular meshwork, the structures responsible for most of the outflow resistance in open angle glaucoma. In this procedure a trabeculo-Descemet's membrane (TDM) is left intact to control aqueous outflow through the filtration site. This controlled pressure reduction is responsible for a better safety profile of DS with lower rate of complications related to over drainage and hypotony. The superior safety profile has been confirmed by several clinical studies and is now wildly accepted.^1^ However, studies show conflicting results for DS efficacy and it continues to be an area of debate. In recent years it has become clear that the use of antimetabolites, implants and Nd:YAG laser goniopuncture greatly improves DS outcome.

## HISTORY OF DEEP SCLERECTOMY

The earliest reports on non penetrating filtering procedures were published in 1964 by Krasnov^2^ and Walker.^3^ In sinusotomy, as described by Krasnov, a band of sclera is removed parallel to the limbus exposing the Schlemm's canal. However, the inner wall of Schlemm's canal and trabecular meshwork were left intact. These procedures never gained popularity as they were difficult to perform without use of the operating microscope, which was not readily available at the time. In late 1960s trabeculectomy, described by Cairns,^4^ became the most common filtering procedure for over four decades.

Non penetrating procedures were further refined in 1980s and 1990s. Zimmermann^5^ and Arenas^6^ advocated the removal of the inner wall of Schlemm's canal with the juxta canalicular trabeculum and the covering of the sclerectomy site with a superficial scleral flap in ab externo trabeculectomy. In DS, described by Fyodorov,^7^ the corneal stroma anterior to the trabecular meshwork and Descemet's membrane is also removed creating a window of TDM as the filtration site.

To improve long term efficacy, DS has been further developed with the use of anti metabolites to reduce postoperative scarring and use of space-maintainer implants to keep the scleral lake open.

## INDICATIONS

Traditional glaucoma teaching places surgery as a procedure of last resort after medical and laser therapies. This is largely due to the relatively high complication rates and unpredictability of trabeculectomy.^8^ DS, with its low complication rates, can be offered at an earlier stage of the disease and even be considered a first line therapy when medical or laser treatment is insufficient or unavailable.

DS targets the site of highest outflow resistance in open angle glaucoma, namely the inner wall of Schlemm's canal and juxtacanalicular trabecular meshwork.^9^ It is indicated in both primary and secondary open angle glaucoma.^10^ DS is also indicated in uveitic glaucoma as it induces less inflammation than penetrating procedures.^11^ High myopes, at higher risk of choroidal detachment, also benefit from the more controlled IOP drop after DS.^12^

Neovascular glaucoma is an absolute contraindication to DS, as a fibro vascular membrane is formed over the irido-corneal angle making filtration through a TDM impossible. Cases of ICE syndrome are contraindicated for the same reason. A narrow angle is a relative contraindication to DS as proximity of the iris can lead to anterior synechia formation or iris incarceration following surgery. DS is also relatively contraindicated in eyes with damaged trabeculum (post traumatic angle recession, post laser trabeculoplasty) as surgical success relies on intact angle structures.^1^

## SURGICAL TECHNIQUE

**Figure 1:**
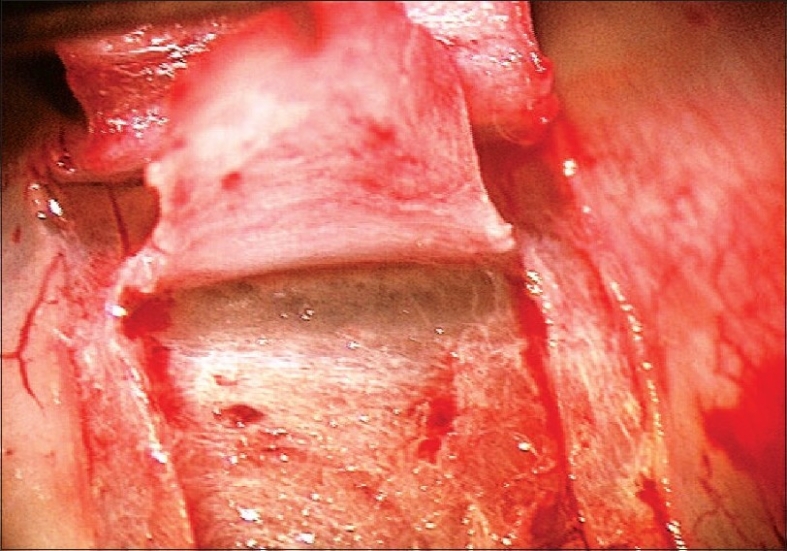
Deroofing of Schlemm's canal

**Figure 2:**
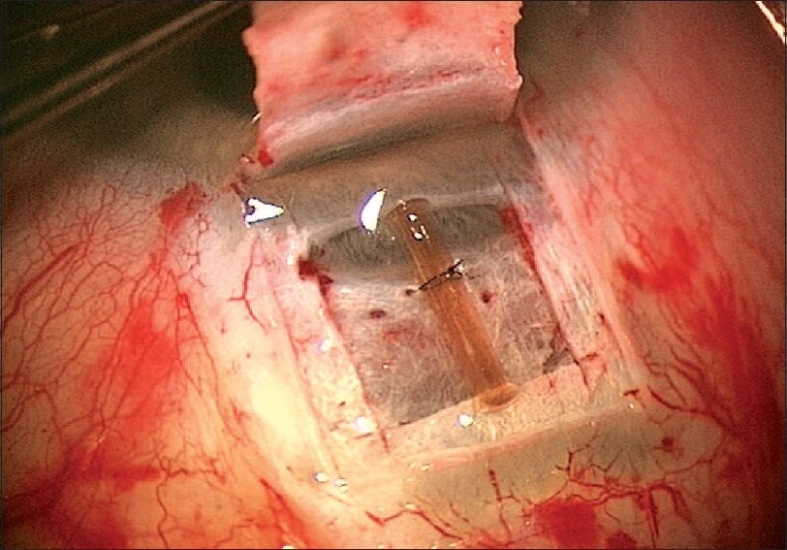
Collagen implant sutured under scleral flap

**Figure 3:**
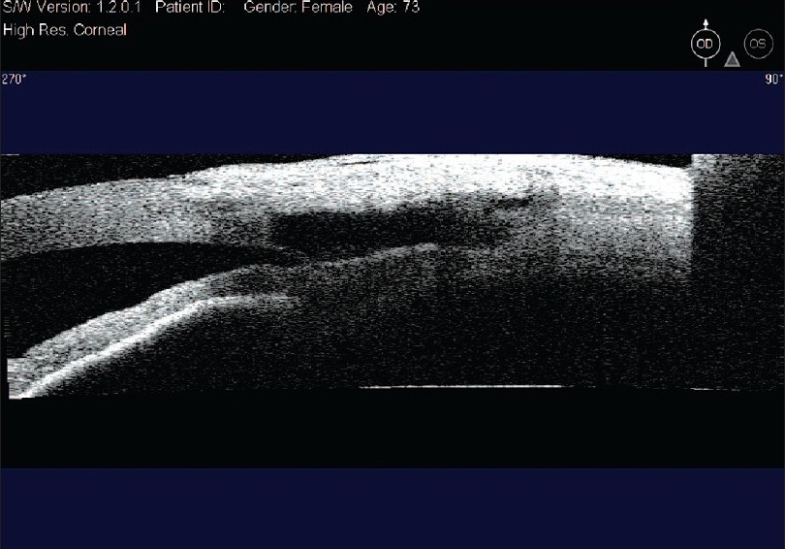
Anterior segment OCT image through surgical site

**Figure 4:**
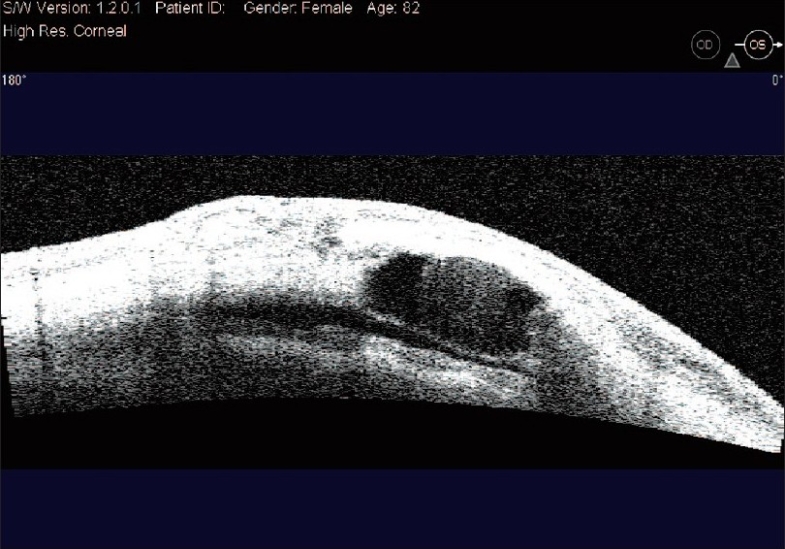
AS-OCT image showing the TDM and collagen implant over the iridocorneal angle

The aim of DS is to create a TDM which functions as site of controlled aqueous outflow as well as to form an intrascleral space to act as a reservoir.^1^ The conjunctiva and Tenon's capsule is opened at the limbus to expose sclera. At this stage, antiproliferatives can be applied to the scleral bed followed by thorough irrigation. A limbus based superficial 5 × 5 mm scleral flap of one-third thickness is fashioned and extended 1.5 mm into clear cornea. A second deep scleral flap is dissected leaving only a 50-100 *μ*m thick scleral bed. This flap should be 4 × 4 mm leaving a step for closure of the superficial flap. Defining the correct tissue plane is critical, as dissecting in this plane Schlemm's canal gets deroofed as the dissection is advanced anteriorly [Figure 1]. The TDM is fashioned by extending the dissection up to 1-1.5 mm into clear cornea. To avoid perforation during this part of the procedure, Descemet's membrane can be gently detached using a sponge or a spatula. The radial incisions extending the deep flap into clear cornea are best done by holding a No 11 steel blade or a 15° diamond knife with the bevel up to avoid entering the anterior chamber. Once this dissection is completed the deep flap is excised using a blade or fine scissors. At this stage aqueous should be percolating through the trabeculum. Once dried, the inner wall of Schlemm's canal and the juxta canalicular trabecular meshwork can be grabbed by fine forceps and peeled off the underlying trabeculum. An implant can be sutured in the scleral bed to act as a space maintainer during the initial healing period, keeping the intrascleral space open [Figure 2]. The superficial scleral flap is then closed with 10/0 nylon sutures. Finally the conjunctiva is closed with 10/0 vicryl sutures. The surgical site can be imaged using the anterior segment OCT, showing the TDM, the scleral lake and the collagen implant [Figures 3 and 4].

## OUTCOME

DS appears to be a safe surgical procedure to reduce IOP. The complication rate appears to be lower; flat anterior chamber, choroidal detachment, hypotonic maculopathy, and postoperative infections are rare.^13^ This is probably due to the controlled IOP decrease through the intact TDM and the absence of a surgical peripheral iridotomy; making deep sclerectomy essentially an extraocular procedure. The efficacy of non penetrating procedures perceived to be inferior compared to trabeculectomy. However, a number of adjunctive techniques are now being used that have been shown to increase efficacy. These include the intraoperative use of antimetabolites, the use of implants as well as performing laser goniopuncture early when the IOP rises above the target value.

DS achieves good IOP control in the early postoperative period. However, without the use of adjunctive therapies an increasing proportion fails over the long term. Khairy *et al*.^14^ in their study of a prospective series of 43 patients undergoing DS without implant or antimetabolite found a success rate (IOP less than 22 mmHg without medication) at 12, 24 and 30 months - 61.4, 36.6, and 18.9% respectively.

The use of implants to maintain the intrascleral space improved the long-term results.^15^ A study by Shaarawy *et al*.^16^ comparing DS in one eye and DS with collagen implant (DSCI) in the other find complete success (IOP less than 21 mmHg without glaucoma medications) at 48 months in 38.5% of DS eyes and 69.2% of eyes after DCSI. Bissig^17^ *et al*. report 10-year postoperative outcomes after DCSI in 105 eyes and find 47.7% complete success (IOP less than 21 mmHg without glaucoma medications) and 88.9% qualified success (IOP less than 21 mmHg with or without glaucoma medications) with laser gonio puncture in 59.8% of patients. Five-fluotouracil (5-FU) injections were required in 24.5% of cases to treat bleb fibrosis or encapsulation.

Several studies show that use of antimetabolites improves success rates. They act by reducing scaring at the filtration site. Kozobolis^18^ *et al.* study compares DS and DS augmented by MMC (0.2 mg/ml/2.5 min) with 36 months follow-up. The use of MMC has been associated with a larger IOP reduction and improved success rate, with 72.5% of DS achieving qualified success versus 95% of DS-MMC. Anand^19^ *et al*. study DS with low dose MMC (0.25 mg/ml/2 min) in a Nigerian population, and fail to show a significant benefit of using an antimetabolite, and had low success rate in both DS and DS MMC groups in this west African population.

Several randomized prospective studies compare DS with trabeculectomy, and most find only small differences in their efficacy to control IOP. El Sayyad *et al.*^20^ perform a paired eye study, randomly assigning 39 patients to have DS or trabeculectomy in one eye and the opposite procedure in the other eye. They found IOP reductions of 15.6 plus/minus 4.2 mmHg in DS and 14.1 plus/minus 6.4 mmHg in trabeculectomy, with a complete success rate (IOP less than 21 no glaucoma meds) of 79% and 85% respectively. Similar results were reported by several other groups.^21^,^22^ Ambersin *et al.*^21^ reported complete success in 40% of DS and 45% of trabeculectomy cases, and a goniopuncture rate of 45% following DS. Cillinio^22^ *et al*. find no significant difference in outcomes between DS and trabeculectomy.

## CONCLUSIONS

DS has evolved over the past decades, and now long term data on its safety and efficacy is becoming available. There is consensus that complications related to over-filtration and infections are significantly lower than in trabeculectomy. The studies summarized above showed that the use of implants, antimetabolites and gonio puncture could ensure good long term IOP control.

The results of El Sayyad from Saudi Arabia are encouraging for the population of that region, however Anand's results from Nigeria were disappointing for a predominantly Afro-Caribbean population due to aggressive scaring. These results are worth bearing in mind when selecting patients for the procedure from different racial backgrounds.

As for any technically challenging procedure the learning curve for DS is long, can take up to 20 cases. However the most common intra-operative complication is TDM perforation, which essentially converts DS to a trabeculectomy, a procedure most surgeons would be familiar with. However we recommend DS for surgeons with a sufficiently high glaucoma surgical caseload that allows for developing and maintaining their technical skills.

The role of DS in the management of open angle glaucoma is still being defined. As it appears to have a superior safety profile than penetrating procedures it could be considered at an earlier stage of the disease, especially in case of medical/laser therapy intolerance or unavailability.

## References

[1] Sarodia U, Shaarawy T, Barton K (2007). Nonpenetrating glaucoma surgery: a critical evaluation. Curr Opin Ophthalmol.

[2] Krasnov MM (1964). Sinusotomy in glaucoma. Vestn Oftalmol.

[3] Walker WM, Kanagasundaram CR (1964). Surgery of the canal of Schlemm. Trans Ophthalmol Soc UK.

[4] Cairns JE (1968). Trabeculectomy. Preliminary report of a new method. Am J Ophthalmol.

[5] Zimmerman TJ, Kooner KS, Ford VJ, Olander KW, Mandlekorn RM, Rawlings EF (1984). Trabeculectomy vs. nonpenetrating trabeculectomy: a retrospective study of two procedures in phakic patients with glaucoma. Ophthalmic Surg.

[6] Arenas E, Boyd B (1994). Trabeculectomy ab-externo. Highlights. World Atlas Series of Ophthalmic Surgery.

[7] Fyodorov SN (1989). Non penetrating deep sclerectomy in open-angle glaucoma. Eye Microsurg.

[8] Watson PG, Jakeman C, Ozturk M, Barnett MF, Barnett F, Khaw KT (1990). The complications of trabeculectomy (a 20-year follow-up). Eye.

[9] Mäepea O, Bill A (1992). Pressures in the juxtacanalicular tissue and Schlemm's canal in monkeys. Exp Eye Res.

[10] Cheng JW, Ma XY, Wei RL (2004). Efficacy of non-penetrating trabecular surgery for open angle glaucoma: a metaanalysis. Chin Med J (Engl).

[11] Mendrinos E, Mermoud A, Shaarawy T (2008). Nonpenetrating glaucoma surgery. Surv Ophthalmol.

[12] Hamel M, Shaarawy T, Mermoud A (2001). Deep sclerectomy with collagen implant in patients with glaucoma and high myopia. J Cataract Refract Surg.

[13] Karlen ME, Sanchez E, Schnyder CC, Sickenberg M, Mermoud A (1999). Deep sclerectomy with collagen implant: medium term results. Br J Ophthalmol.

[14] Khairy HA, Green FD, Nassar MK, Azuara-Blanco A (2006). Control of intraocular pressure after deep sclerectomy. Eye.

[15] Sanchez E, Schnyder CC, Sickenberg M, Chiou AG, Hédiguer SE, Mermoud A (1996). Deep sclerectomy: results with and without collagen implant. Int Ophthalmol.

[16] Shaarawy T, Mermoud A (2005). Deep sclerectomy in one eye vs deep sclerectomy with collagen implant in the contralateral eye of the same patient: long-term follow-up. Eye.

[17] Bissig A, Rivier D, Zaninetti M, Shaarawy T, Mermoud A, Roy S (2008). Ten years follow-up after deep sclerectomy with collagen implant. J Glaucoma.

[18] Kozobolis VP, Christodoulakis EV, Tzanakis N, Zacharopoulos I, Pallikaris IG (2002). Primary deep sclerectomy versus primary deep sclerectomy with the use of mitomycin C in primary open-angle glaucoma. J Glaucoma.

[19] Mielke C, Dawda VK, Anand N (2006). Deep sclerectomy and low dose mitomycin C: a randomised prospective trial in West Africa. Br J Ophthalmol.

[20] El Sayyad F, Helal M, El-Kholify H, Khalil M, El-Maghraby A (2000). Nonpenetrating deep sclerectomy versus trabeculectomy in bilateral primary open-angle glaucoma. Ophthalmology.

[21] Ambresin A, Shaarawy T, Mermoud A (2002). Deep sclerectomy with collagen implant in one eye compared with trabeculectomy in the other eye of the same patient. J Glaucoma.

[22] Cillino S, Di Pace F, Casuccio A, Calvaruso L, Morreale D, Vadalà M (2004). Deep sclerectomy versus punch trabeculectomy with and without phacoemulsification: a randomised clinical trial. J Glaucoma.

